# Influence of Geographical Area and Living Setting on Children's Weight Status, Motor Coordination, and Physical Activity

**DOI:** 10.3389/fped.2021.794284

**Published:** 2022-01-20

**Authors:** Maria Chiara Gallotta, Giovanna Zimatore, Lavinia Falcioni, Silvia Migliaccio, Massimo Lanza, Federico Schena, Valentina Biino, Matteo Giuriato, Marianna Bellafiore, Antonio Palma, Giuseppe Battaglia, Carlo Baldari, Laura Guidetti

**Affiliations:** ^1^Department of Physiology and Pharmacology “Vittorio Erspamer”, Sapienza University of Rome, Rome, Italy; ^2^Department of Theoretical and Applied Sciences, eCampus University, Novedrate, Italy; ^3^CNR (National Research Council) Institute for Microelectronics and Microsystems, Bologna, Italy; ^4^Department of Movement, Human and Health Sciences, University of “Foro Italico”, Rome, Italy; ^5^Department of Neuroscience, Biomedicine and Movement, University of Verona, Verona, Italy; ^6^Department of Human Sciences, University of Verona, Verona, Italy; ^7^Department of Psychology, Educational Science and Human Movement, University of Palermo, Palermo, Italy; ^8^Department of Unicusano, University Niccolò Cusano, Rome, Italy

**Keywords:** overweight, obesity, movement skills, gender, geographical area, living setting, health cities

## Abstract

This study was aimed (i) to examine the effect of living setting (rural vs. urban), geographical area (North vs. Center vs. South), and gender (boys vs. girls) on weight status, motor coordination, and physical activity (PA) level of Italian school-age children; (ii) to examine differences in the neighborhood walkability of different school areas from different geographical areas and living settings; and (iii) to examine whether motor coordination, PA level, geographical areas, living setting, neighborhood walkability, and gender could predict children's weight status. We assessed anthropometric parameters, gross motor coordination, and PA level in 1,549 children aged between 8 and 13 years. Results revealed that Central children had higher BMI than Northern and Southern children (η^2^ = 0.01). Moreover, Northern children showed the highest motor quotient (η^2^ = 0.148) and PA level (η^2^ = 0.02), followed by Southern and Central children, respectively. Children from the South of Italy attended schools located in neighborhoods with the highest Walk Score®. Urban children attended schools located in neighborhoods with a higher Walk Score® than rural children. Lower motor quotient (MQ), lower PA level, and living in a rural setting and in a car-dependent neighborhood were associated with a higher relative risk for obesity. Being a girl was associated with a lower relative risk for obesity. The alarming high percentage of overweight and obesity in children as well as motor coordination impairments revealed the urgent need of targeted PA interventions in pediatric population.

## Introduction

The prevalence of overweight and obesity in childhood is increasing at an alarming rate worldwide, particularly in industrialized countries. The excess of body weight is strongly correlated with sedentary lifestyles and therefore is related to low levels of motor competence (1, 2). It can be defined as mastery in fundamental movement skills such as walking, running, or jumping and in more specialized movement sequences such as lifelong physical activity abilities like cycling, swimming, or sport-specific skills, and it describes the ability to perform both gross and fine motor skills (3). Studies reported a substantial decline in children's motor competence over the last 4 decades due to the decline of physical activity (PA) and the increase of sedentary behaviors (4). Interestingly, recent studies conducted on Italian populations report that more than 30% of Italian children are affected by overweight or obesity (5, 6), 18% are sedentary, while 41% perform more than 2 h of screen activities a day. Sallis and Glanz identified several environmental and demographic variables affecting children's PA level and obesity (7). Urban and rural settings appear as important conditioning factors for participation in PA and for the development of fitness and coordination (8). Studies have been conducted to identify the association between the PA of children and adolescents and the setting in which they live (7, 9). The ease of access to safe and outdoor sites promotes PA in children, who therefore improve their physical fitness and coordinative abilities (1, 10). Contrarily, the lack of sidewalks and recreational facilities, the absence of ease of access to schools, the need to cross busy streets, traffic congestion, and air pollution discourage children from playing outside or from walking and biking to school (7, 10, 11), favoring an obesogenic environment (12). Therefore, PA, obesity levels, and the associated motor competence during childhood, might correlate with the level of urbanization. Recent studies investigated the influence of the living setting on anthropometric parameters, PA level, and motor competence in children, with inconsistent and contrasting results (13, 14). No consensus exists concerning a definition of residential areas in terms of urban and rural specificity since most studies define urban and rural setting only on population density (14). Furthermore, obesity in childhood could be influenced by the spatial structure of street networks and by the aspects of the built environment (15) that modify neighborhood walkability and, thus, PA levels (16). The Italian peninsula, mostly within the Apennine mountain range, stretches for about 1,200 km, in NW–SE striking sets leading to many different historical and geographical characteristics that determine significative socio-economic and lifestyle differences among northern, central, and southern regions and, also, between urban and rural settings (17).

Considering the scientific evidence reported, we hypothesized that the geographical area and living setting could influence the weight status, motor coordination, and PA level of Italian school-age children. Therefore, the first aim of the present cross-sectional study was to examine differences in weight status, motor coordination, and PA level between boys and girls from different geographical areas (North vs. Center vs. South) and living settings (rural vs. urban). The second aim was to examine differences in the neighborhood walkability of different school areas from different geographical areas and living settings. Finally, the third aim was to examine whether motor coordination, PA level, geographical areas, living setting, neighborhood walkability, and gender could predict children's weight status.

## Materials and Methods

### Participants

An open invitation to representatives of private and public schools of the Italian geographical areas (North Italy = Veneto and Trentino-Alto Adige; Center Italy = Lazio; South Italy = Sicily) was done, and a total sample of 2,206 schoolchildren were recruited after school principals/administrators had been informed about the whole project and accepted to participate in the study. A final sample of 1,149 schoolchildren aged between 8 and 13 years volunteered to participate in this study and completed all measurements. The population included subjects from 38 different Italian primary and middle schools. The classroom demographics broke down to 391 grade 3 children (8–9 years of age), 362 grade 4 children (9–10 years of age), 351 grade 5 children (10–11 years of age), 234 grade 6 children (11–12 years of age), and 211 grade 7 children (12–13 years of age). The participating schools were enrolled to be broadly representative of Northern, Central, and Southern schools, including the capital city (Rome) and the urban and rural areas, and to have appropriate and similar sports facilities to conduct comparable measurements. The measurements of this study were conducted in the participating schools from January 2019 to February 2020 during the regular school hours and in the respective school gyms.

The University Ethical Committees of the University of Rome (Rif 5500 Prot. 1070/19), of the University of Verona (No. 2019-UNVRCLE-0298910), and of the University of Palermo (No. 8/2019) in accordance with the ethical standards laid down in the 1964 Declaration of Helsinki and its later amendments. Additional authorization was provided by school principals/administrators. Written informed consent forms were obtained from parents prior to study participation.

### Anthropometric Measurements

As for the anthropometric measurements, children's body weight and height were collected. Anthropometric measurements were taken according to the standard procedures described by the International Society for the Advancement of Kinanthropometry (18). Children's body weight and height were measured using a scale and a stadiometer to the nearest 0.5 kg and 0.1 cm, respectively. Subjects wore minimal clothing and were barefoot. Body mass index (BMI) was calculated as weight in kilograms divided by the square of height in meters. All measures were collected by examiners who were trained in the measurement methods of height and weight. Children were classified as underweight (UW), normal weight (NW), overweight (OW), and obese (OB) using age- and gender-specific International Obesity Task Force cut-off points (19).

### Gross Motor Coordination Measurement

Gross motor coordination was assessed by the Körperkoordinations Test für Kinder (Body Coordination Test for Children, referred to as KTK) battery (20), consisting of the following subtests:

- walking backward three times along each of three balance beams (3-m length; 6-, 4.5-, and 3-cm width, respectively; 5-cm height). A maximum of 24 steps (eight per trail) were counted for each balance beam, yielding to a maximum of 72 steps (24 steps × 3 beams) for this test.
- moving across the floor in 20 s by stepping from one plate (25 × 25 × 5.7 cm) to the next, transferring the first plate, stepping on it, etc. The number of relocations was counted and summed over two trials.
- jumping from one leg over an increasing pile of pillows (60 × 20 × 5 cm each) after a short run-up. Three, two, or one point(s) were/was awarded for successful performance on the first, second, third trials, respectively. A maximum of 39 points (ground level + 12 pillows) could be scored for each leg, yielding a possible maximum score of 78.
- jumping laterally as many times as possible over a wooden slat (60 × 4 × 2 cm) in 15 s. The number of jumps over two trials was summed.

The test–retest reliability coefficient for the raw score on the total test battery was reported as 0.97, while corresponding coefficients for individual tests ranged from 0.80 to 0.96. Both factor analysis and inter-correlations indicated acceptable construct validity (20).

The raw test scores from each of the four subtests were then transformed into gender- and age-specific motor quotient (MQ) values, which were based on the performance of 1,228 normally developing German children. Scoring of the KTK test was performed according to the manual (20). MQ is a global indicator of gross motor coordination, and values between 86 and 115 describe the normality (20).

In the present study, children were categorized as “children with MQ impairments” (MQ score ≤ 85) or “children with no MQ impairments” (MQ score ≥ 86) (20).

### Physical Activity Level Measurements

The Italian version of the Physical Activity Questionnaire for Older Children (PAQ-C-It) was adopted to measure the children PA level (21). This instrument is a valid and reliable self-administered, 7-day recall instrument designed to measure general levels of PA in children aged 8–14 years. The questionnaire consists of nine questions about sports and games, physical activities at school, and those during leisure time that a child might have done in the last 7 days, including the weekend. Each question is scored from 1 (low PA) to 5 (high PA), with the final score obtained through the means of the question scores. The final score represents the activity level of the child (21). Three different activity levels were defined according to Chen's specific cut-offs (22): low ( ≤ 2), moderate (>2 and ≤ 3), and high activity (>3) level. For the present study, children were subdivided into “inactive” (PA score ≤ 2) or “active” (PA score > 2) (22).

### Geographical Area and Living Setting

Three different geographical areas were considered: North, Center, and South of Italy. Moreover, two different settings were considered: urban and rural settings, defined by population density (www.reterurale.it). According to this classification, urban areas have a population density higher than 150 inhabitants/km^2^ and rural areas have a population density lower than 150 inhabitants/km^2^ (23). Population density was determined according to the most recent data provided by ISTAT (Istituto Nazionale di Statistica, Census, www.istat.it).

Seven hundred and seventy-two children came from the North of Italy (535 urban and 237 rural children), 411 children came from the Center of Italy (181 urban and 230 rural children), and 366 children came from the South of Italy (268 urban and 98 rural children) (Table 1).

**Table 1:** Characteristics of the subjects by geographical area, living setting, and gender (*n* = 1,549).

	**North** **(*****n*** **=** **772)**				**Center** **(*****n*** **=** **411)**				**South** **(*****n*** **=** **366)**			
	**Rural** **(*****n*** **=** **237)**		**Urban** **(*****n*** **=** **535)**		**Rural** **(*****n*** **=** **230)**		**Urban** **(*****n*** **=** **181)**		**Rural** **(*****n*****=** **98)**		**Urban** **(*****n*** **=** **268)**	
	**Boys** ** (125)**	**Girls** ** (112)**	**Boys** ** (270)**	**Girls** ** (265)**	**Boys** ** (130)**	**Girls** ** (100)**	**Boys** ** (97)**	**Girls** ** (84)**	**Boys** ** (54)**	**Girls** ** (44)**	**Boys** ** (143)**	**Girls** ** (125)**
Age (years)	10.9 ± 1.4	10.4 ± 1.4	10.4 ± 1.6	10.4 ± 1.7	9.5 ± 1.0	9.4 ± 0.9	10.7 ± 1.3	10.5 ± 1.2	10.5 ± 1.3	10.2 ± 1.0	10.3 ± 1.4	10.2 ± 1.4
Weight (kg)	40.2 ± 9.8	39.3 ± 9.6	37.9 ± 9.9	36.8 ± 10.8	37.9 ± 10.2	37.3 ± 11.2	40.9 ± 11.1	39.2 ± 11.6	40.2 ± 11.6	35.6 ± 7.4	39.5 ± 11.6	38.5 ± 10.4
Height (cm)	145.1 ± 10.1	141.3 ± 9.2	143.4 ± 11.2	141.6 ± 12.1	138.4 ± 7.3	137.1 ± 9.0	144.1 ± 11.0	142.5 ± 11.0	146.5 ± 10.3	143.8 ± 7.8	144.4 ± 11.0	143.0 ± 10.2
BMI (kg/m^2^)	19.0 ± 3.3	19.5 ± 3.7	18.2 ± 3.0	18.0 ± 3.2	19.6 ± 4.2	19.5 ± 4.2	19.4 ± 3.2	19.0 ± 3.9	18.5 ± 4.0	17.2 ± 3.1	18.6 ± 3.6	18.6 ± 3.4
MQ (scores)	96.1 ± 16.0	92.4 ± 13.6	92.8 ± 15.6	86.9 ± 15.5	82.1 ± 11.8	76.2 ± 12.5	78.6 ± 13.8	76.5 ± 13.1	81.8 ± 13.9	70.6 ± 11.8	86.1 ± 17.0	81.0 ± 14.8
PA level (scores)	2.7 ± 0.6	2.6 ± 0.4	2.6 ± 0.5	2.6 ± 0.4	2.4 ± 0.7	2.0 ± 0.6	2.5 ± 0.9	2.4 ± 0.7	2.6 ± 0.6	2.5 ± 0.6	2.4 ± 0.6	2.3 ± 0.6

*Data are expressed as mean ± SD*.

*BMI, body mass index; MQ, motor quotient; PA, physical activity*.

Northern-urban children comprised pupils attending 14 different schools located in Bolzano, in Verona, and in the provinces of Treviso (Castello di Godego), of Verona (Lugagnano, San Bonifacio, Bovolone, Castelnuovo del Garda, and Mozzecane), of Mantova (San Giorgio Bigarello), and of Padua (Casale di Scodosia), while northern-rural children included pupils attending seven different schools located in the province of Verona (Roverchiara, Casaleone, Minerbe, and Poiano) and of Padua (Castelbaldo, Masi, and Merlara). Central-urban children comprised pupils attending six different schools located in Rome, while central-rural children included pupils attending four different schools located in the province of Rome (Montelibretti, Montorio Romano, Monteflavio, and Nerola).

Southern-urban children included pupils attending six different schools located in Palermo and in the province of Palermo (Villabate), while southern-rural children included pupils attending one school in the province of Messina (Mistretta).

### Neighborhood Walkability of School Areas

Walkability measurements of the different school areas were collected using the free open software Walk Score® (WS; www.walkscore.com). Walk Score® is a valid measure of estimating neighborhood walkability in many geographic locations (24). Each child's school address was manually entered into Walk Score®, and from here the walkability of the different school areas was analyzed. For each address, Walk Score® calculates all the different walking routes to nearby amenities (public transit stations, grocery stores, retail stores, parks, schools) producing, through an algorithm, a score ranging from 0 to 100. A score of 100 is assigned to districts that have amenities within a 5-min walk (400 m), whereas areas with more distant amenities report a lower score, with a 0 score given after a 30-min address-amenities walking (when the amenities are more distant than 1 mile).

In the present study, the different school areas were categorized as “walkable area” (50–100 scores) or “car-dependent area” (0–49 scores) (www.walkscore.com).

### Statistical Analysis

General characteristics of the total group and for boys and girls as well as for urban and rural residents and for Northern, Central, and Southern residents were described by means, standard deviations, and frequencies. The chi-square test was used to compare the frequencies of variables among groups (gender, geographical area, and living setting). The chi-square test was also used to compare the frequencies of children attending schools located in walkable and “car-dependent” areas between groups (gender, geographical area, and living setting).

ANOVA was performed to examine differences and interactions on BMI (kg/m^2^), MQ (scores), and PA levels (scores) between boys and girls from different geographical areas and living settings.

ANOVA was also performed to examine differences in neighborhood walkability (Walk Score®) of different school areas from different geographical areas and living settings. These analyses were followed by *post-hoc* analysis (Bonferroni adjustment) when significant main effects or interactions were observed. Effect size was also calculated using Cohen's definition of small, medium, and large effect size (as partial η^2^ = 0.01, 0.06, 0.14, respectively) (25).

A multinomial logistic regression analysis was used to assess whether MQ (scores), PA level (scores), geographical area (North vs. Center vs. South), living setting (rural vs. urban), walkability (“car-dependent area” vs. “walkable area”), and gender (boys vs. girls) predicted BMI categories. Underweight and normal weight children were combined as “NW_UW category,” which was set as the reference group. Geographical area, living setting, walkability, and gender were added as factors, MQ and PA level were included in the analyses as covariates. All variables were tested in the same model, controlling the effect of each other. Statistical significance was set at *p* ≤ 0.05, and all analyses were performed using IBM SPSS statistics version 25.

## Results

### Characteristics of the Population

Characteristics of the subjects by gender, geographical area, and living setting are shown in Table 1.

Globally, the results demonstrated that the prevalence of overweight and obesity was 22.0% (*n* = 303 children) and 9.9% (*n* = 136 children), respectively (sample *n* = 1,377). Within the overweight category, 171 children were boys (56.4%) and 132 were girls (43.6%); 122 were Northern (40.3%), 98 were Central (32.3%), and 83 were Southern (27.4%) children; and 115 were rural (38.0%) and 188 were urban (62.0%) children. Within the obesity category, 70 children were boys (51.5%) and 66 girls (48.5%); 43 were Northern (31.6%), 65 were Central (47.8%), and 28 were Southern (20.6%) children; and 76 were rural (55.9%) and 60 were urban (44.1%) children. The chi-square test detected that the proportion of OW_OB children was different between the three geographical regions (North 27.1% vs. Center 39.9% vs. South 30.8%, *p* < 0.001) and the two living settings (rural 37.2% vs. urban 28.7%, *p* = 0.001).

Regarding gross motor coordination, 47.9% of the total sample (*n* = 1,549) showed MQ impairments (*n* = 742). Of these children, 352 were boys (47.4%) and 390 were girls (52.6%). Moreover, 259 were Northern (34.9%), 274 were Central (36.9%), and 209 were Southern (28.2%) children; 272 were rural (36.7%) and 470 were urban (63.3%) children. The chi-square test revealed that the proportion of children with MQ impairments was different among the three geographical regions (North 33.5% vs. Center 66.7% vs. South 57.1%, *p* < 0.001). In addition, there was a higher proportion of MQ impairments in girls than in boys (53.4 vs. 43.0%, respectively, <0.001).

Regarding PA level, 29.0% of the children (*n* = 793) were inactive (*n* = 230): 106 were boys (46.1%) and 124 were girls (53.9 %); 14 were Northern (6.1%), 116 were Central (50.4%), and 100 were Southern (43.5%) children; and 114 were rural (49.6%) and 116 were urban (50.4%) children. The chi-square test detected that the proportion of inactive children was different between the three geographical regions (North 9.6% vs. Center 38.0% vs. South 29.3%, *p* < 0.001). Furthermore, there was a higher proportion of inactive children in girls than in boys (34.1 vs. 24.8%, respectively, *p* = 0.004).

Finally, the chi-square test revealed that the proportion of children attending schools located in “car-dependent” areas is different among the geographical regions (North 36.9% vs. Center 56.0% vs. South 0%, *p* < 0.001). Moreover, there was a higher proportion of “car-dependent areas” in rural as compared to urban schools (79.8 vs. 6.5%, respectively, *p* < 0.001).

### Differences of Gender, Geographical Area, and Living Setting on BMI

Differences for geographical area (*F*_1,1365_ = 9.62, *p* < 0.001, η^2^ = 0.01) revealed that children from Central Italy had higher BMI than Northern and Southern children (19.4 ± 3.9 vs. 18.5 ± 3.3 vs. 18.4 ± 3.5 kg/m^2^, respectively).

**Figure 1:**
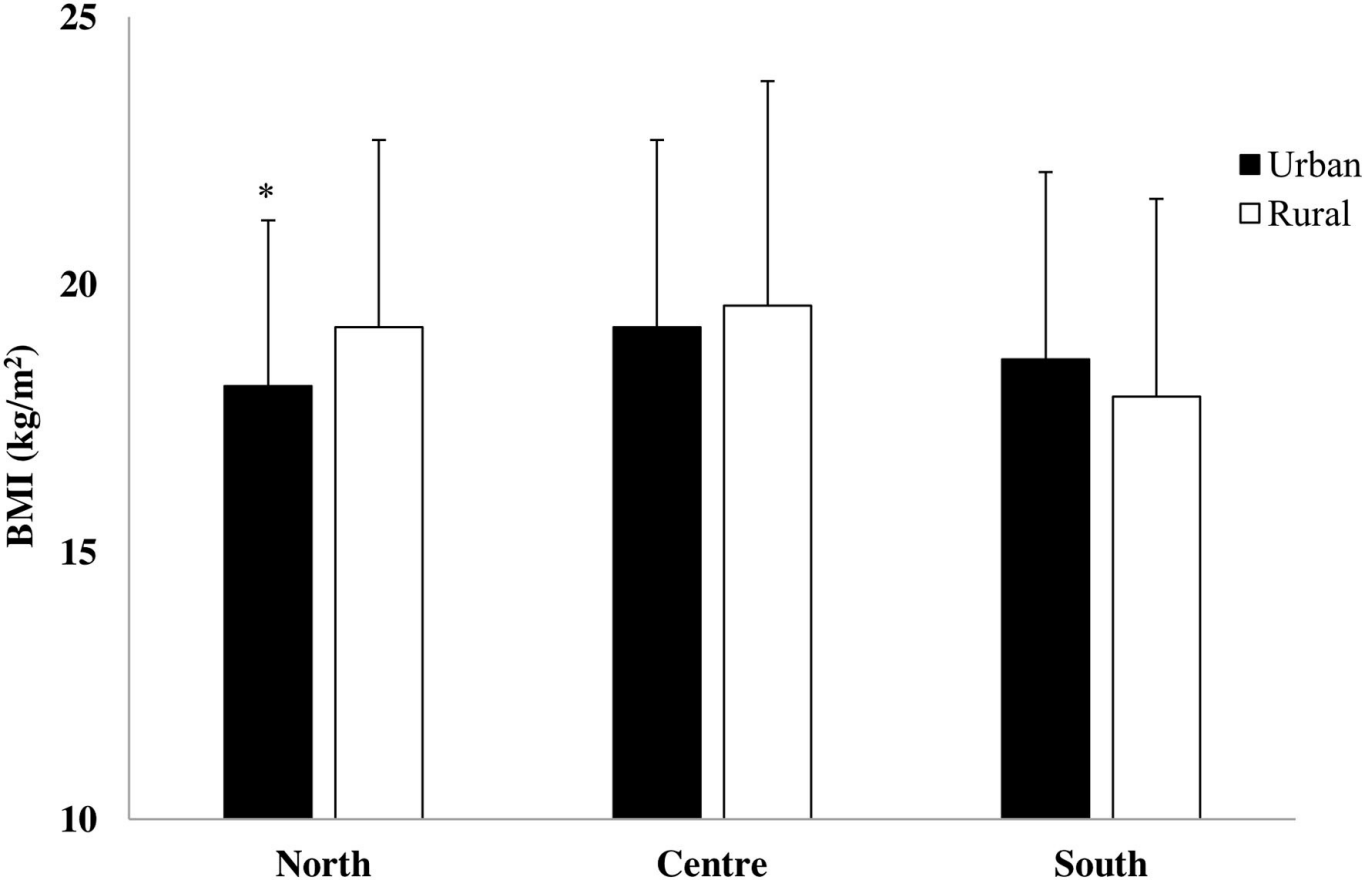
Body mass index (BMI) in urban and rural children of the North, Center, and South of Italy (*p = 0.0001 urban vs. rural).

Interestingly, geographical area x living setting interaction (*F*_2,1365_ = 6.51, *p* = 0.002, η^2^ = 0.01) showed that in the North of Italy, rural children had higher BMI than urban children (Figure 1).

### Differences of Gender, Geographical Area, and Living Setting on Gross Motor Coordination (MQ)

Differences for gender (*F*_1,1537_ = 43.86, *p* < 0.001, η^2^ = 0.03) revealed that boys had higher MQ than girls (88.0 ± 16.2 vs. 83.1 ± 15.6 scores, respectively).

Differences for geographical area (*F*_2,1537_ = 133.14, *p* < 0.001, η^2^ = 0.148) showed that Northern children had the highest MQ, followed by Southern and Central children (91.3 ± 15.7 vs. 81.8 ± 15.9 vs. 78.7 ± 12.9 scores, respectively).

**Figure 2:**
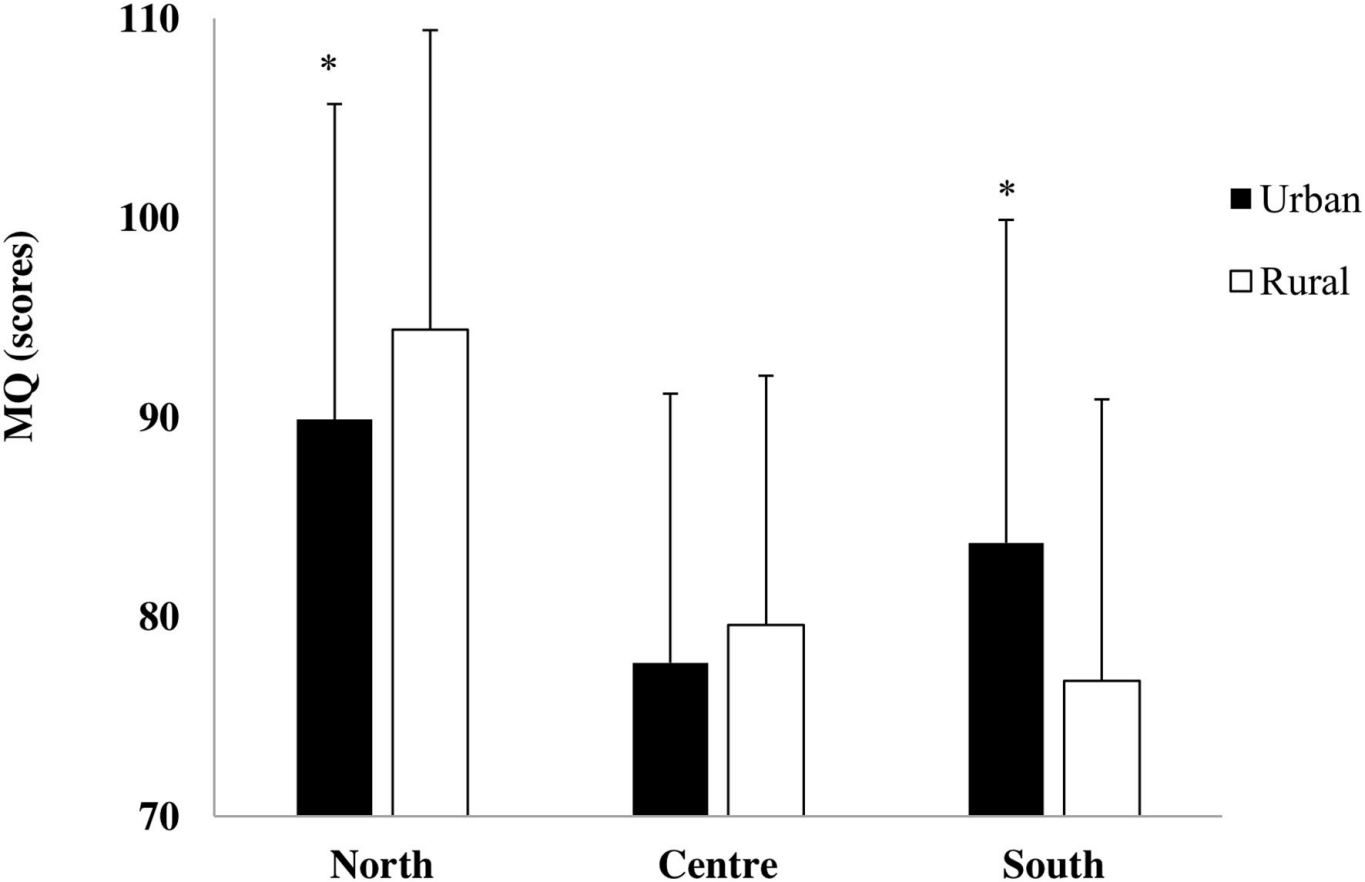
Motor quotient (MQ) in urban and rural children of the North, Center, and South of Italy (*p = 0.0002 urban vs. rural).

Geographical area x living setting interaction (*F*_2,1537_ = 15.88, <0.001, η^2^ = 0.02) showed that in the North of Italy, rural children had a higher MQ than urban children, while in the South of Italy, urban children had a higher MQ than rural children (Figure 2).

### Differences of Gender, Geographical Area, and Living Setting on PA Level

Differences for gender (*F*_1,780_ = 6.03, *p* = 0.014, η^2^ = 0.01) revealed that boys had a higher PA level than girls (2.5 ± 0.7 vs. 2.3 ± 0.6 scores, respectively).

Differences for geographical area (*F*_2,780_ = 7.39, *p* = 0.001, η^2^ = 0.02) showed that Northern children had the highest PA level, followed by Southern children and then Central children (2.6 ± 0.4 vs. 2.4 ± 0.6 vs. 2.3 ± 0.8 scores, respectively).

**Figure 3:**
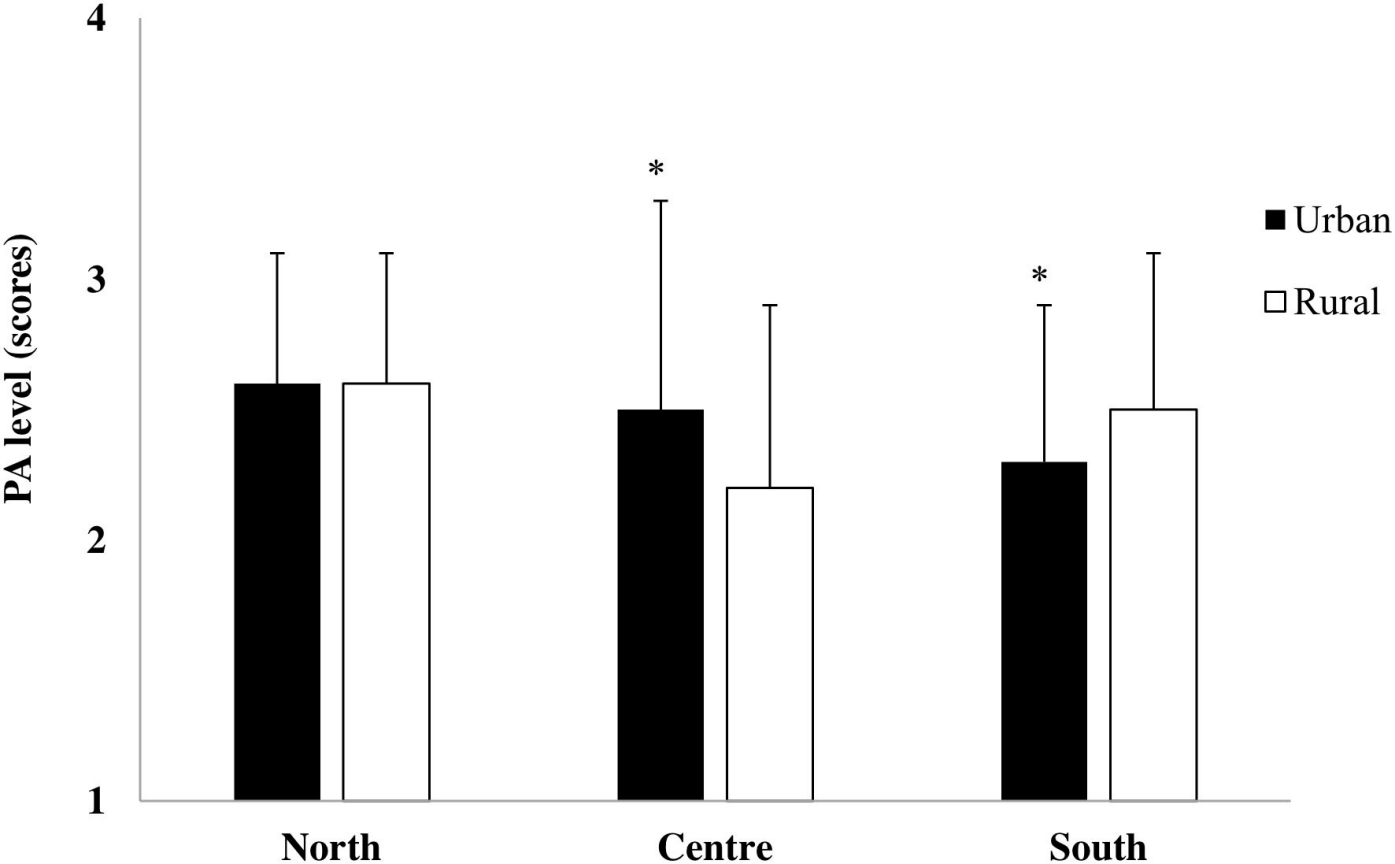
Physical activity (PA) level in urban and rural children of the North, Center, and South of Italy (*p ≤ 0.01 urban vs. rural).

Geographical area x living section interaction (*F*_2,780_ = 9.12, *p* < 0.001, η^2^ = 0.02) showed that in the Center of Italy, urban children had a higher PA level than rural children, while in the South of Italy, rural children had a higher PA level than urban children (Figure 3).

### Differences of Geographical Area and Living Setting on Neighborhood Walkability

Differences for geographical area (*F*_2,1543_ = 170.76, *p* < 0.001, η^2^ = 0.18) revealed that children from the South of Italy attended schools located in neighborhoods with the highest Walk Score®, followed by children from the North and then by children from the Center of Italy (75.7 ± 16.6 vs. 61.6 ± 18.8 vs. 59.4 ± 28.8 score, respectively).

Differences for living setting (*F*_1,1543_ = 4,304.10, *p* < 0.001, η^2^ = 0.74) showed that urban children attended schools located in neighborhoods with a higher Walk Score® than rural children (78.4 ± 13.5 vs. 39.9 ± 11.1 score, respectively).

**Figure 4:**
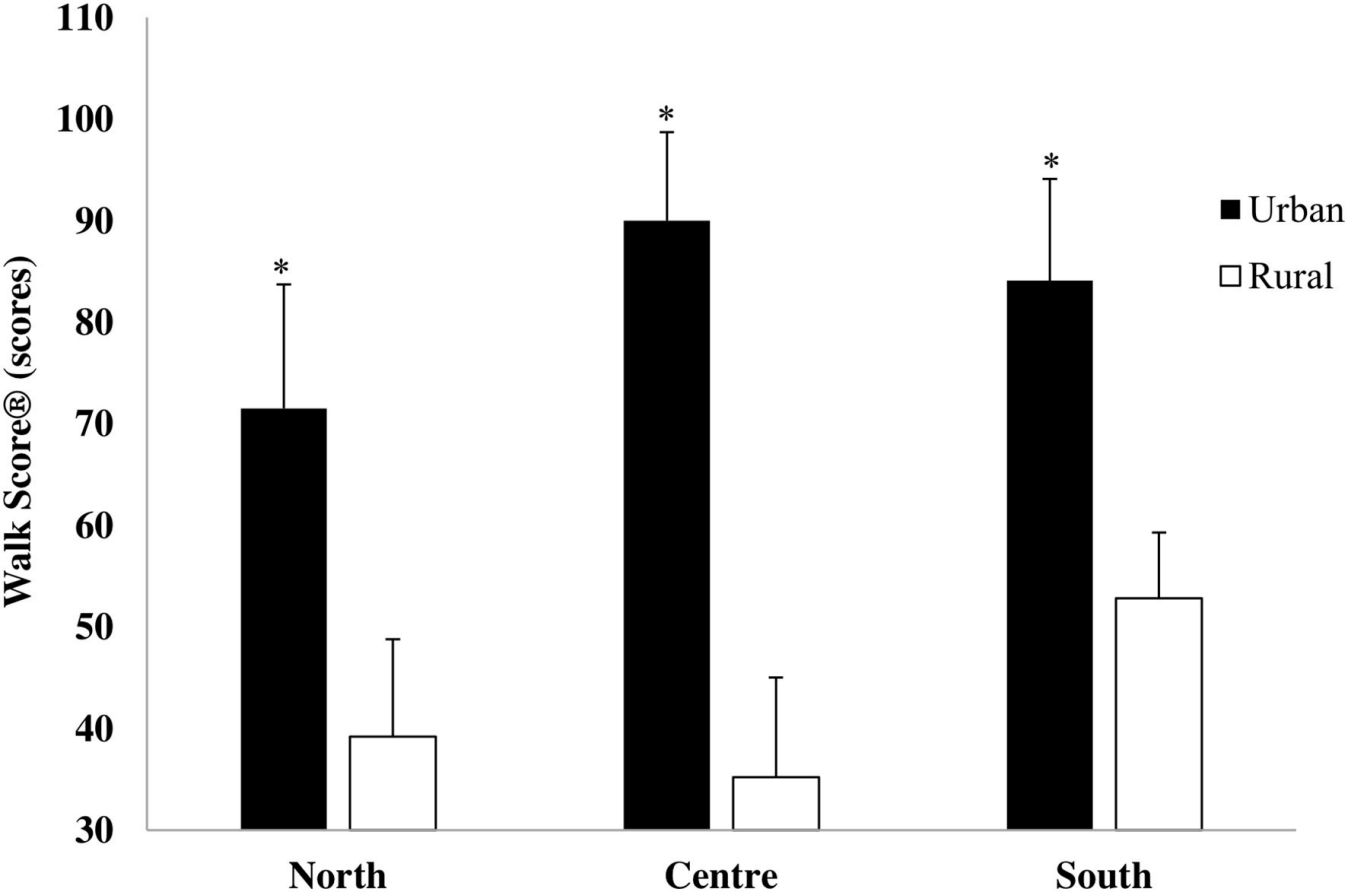
Walk Score® in urban and rural children of the North, Center, and South of Italy (*p < 0.0001 urban vs. rural).

Geographical area x living setting interaction (*F*_1,1543_ = 167.68, *p* < 0.001, η^2^ = 0.18) revealed that in the North, the Center, and the South of Italy, rural children attended schools located in neighborhoods with a lower Walk Score® than urban children (Figure 4).

### Determinants of BMI Categories

In order to model the relationship between BMI categories and several potential predictors (MQ, PA level, living setting, geographical area, walkability, and gender), a multinomial logistic regression was performed. MQ [$\chi_{\left(2\right)}^{2}$ = 93.54, *p* < 0.001], PA level, [$\chi_{\left(2\right)}^{2}$ = 5.77, *p* = 0.056], living setting [$\chi_{\left(2\right)}^{2}$ = 7.19, *p* = 0.027], gender [$\chi_{\left(2\right)}^{2}$ = 10.58, *p* = 0.005], and walkability [$\chi_{\left(2\right)}^{2}$ = 11.28, *p* = 0.004] contributed significantly to the model. Table 2 presents the results of the multinomial logistic regression. Lower MQ was associated with a higher risk for being affected by overweight and obesity. Lower PA level was associated with a higher risk for being obese, and girls showed a lower risk for being obese. Interestingly, living in a rural setting was associated with a higher risk for overweight and obesity and living in a “car-dependent” area was associated with a higher risk for obesity. Finally, living in North, Center, or South of Italy did not predict the BMI categories.

**Table 2:** Multinomial logistic regression predicting BMI categories.

	**OW**	**OB**
	**OR (95% CI)**	**OR (95% CI)**
Gender^a^		
Girls	0.70 (0.48–1.02)	0.47 (0.29–0.77)***
MQ (score)	0.96 (0.94–0.97)****	0.93 (0.91–0.95)****
PA level (score)	0.75 (0.56–1.01)	0.69 (0.47–1.01)*
Geographical area^b^		
North area	0.95 (0.54–1.68)	0.88 (0.37–2.08)
Center area	0.79 (0.44–1.42)	0.91 (0.41–2.02)
Living setting^c^		
Rural setting	0.54 (0.29–1.02)*	0.33 (0.12–0.95)**
Walkability^d^		
Car-dependent neighborhood	2.16 (0.93–5.00)	6.89 (1.94–24.43)***

*NW_UW was chosen as the reference group for the outcome*.

a *Reference category is “boys.”*

b *Reference category is “South area.”*

c *Reference category is “urban setting.”*

d *Reference category is “walkable neighborhood.”*

*All variables were tested in the same model, controlling the effect of each other*.

*p = 0.05, **p < 0.05, ***p ≤ 0.01, ****p < 0.0001.

*OW, overweight; OB, obese; NW_UN, normal weight and underweight; OR, odds ratio; CI, confidence interval; MQ, motor quotient*.

## Discussion

The first aim of the present study was to examine differences in weight status, motor coordination, and PA level between boys and girls from different geographical areas and living settings. The hypothesis we formulated was confirmed because our findings showed that children from the Center of Italy had a higher BMI than their peers from the North and the South, revealing the higher proportion of overweight and obese children in the Italian Central regions. These results were not in line with previous research findings that reported the higher prevalence of children's and adolescents' overweight and obesity in the South regions of Italy when compared with the Center and the North regions (5, 6, 17, 26). Our Central children also showed the lowest PA level and the worst walkability of neighborhoods when compared with their Northern and Southern peers, which could have negatively affected their weight status. Moreover, the greater BMI of children living in rural areas of North Italy was consistent with results reported for children living in rural areas of Midwest in the United States (27) and for children living in rural areas of Croatia (13). In addition, considering the different weight status categories, it appeared that rural children had higher overweight/obesity prevalence than urban children, underlining the severe situation of youth living in this setting. Although in the present study socio-economic factors were not measured, rural children were often associated with a low family income (28). Therefore, we could speculate that this low socio-economic status of rural children leads to an unhealthy lifestyle, which is directly related to low levels of PA, to an unhealthy diet (29), and to a high prevalence of overweight and obesity (17).

Our results revealed a higher prevalence of motor impairments in girls than in boys, indicating that boys at comparable ages are more coordinated than girls. Similar results, previously observed in Portuguese children, suggested that these differences could be due to different motor skills refinements, body growth, and physical fitness levels between boys and girls (30). This significant difference between the MQ of boys and girls could also be explained by referring to gender stereotypes in PA and sport practice (31, 32). Sport (i.e., football, athletics, basketball) has a strong masculine connotation, probably favoring males' participation and practice in out-of-school settings and therefore their higher performance in motor tests (31, 32). Moreover, our results revealed that boys had a higher PA level than girls. The higher prevalence of physical inactivity among girls was consistent with results reported by other studies (5, 29). The low levels of gross motor coordination in combination with low levels of PA in girls suggest that this population needs to be targeted for priority intervention programs to promote PA and sport participation in girls.

Northern children showed better gross motor coordination level when compared with Central and Southern children. These results could be explained by good leisure time facilities and the strong emphasis to promote exercise and sport practice in many Northern municipalities (5), thus providing an environment that could promote children's active behaviors. In fact, Northern children were the most active, showing a higher PA level than Southern and Central children. Contrarily, Central areas had more barriers to PA due to the lack of safety, green spaces, sports facilities, and walkable neighborhoods that could determine the worst MQ scores of children from the Center of Italy (Figure 2) (33). Moreover, our Northern rural children had a higher MQ than their urban peers, showing an opposite scenario in the South of Italy, where urban children had a higher MQ than their rural peers. These controversial results were in line with results reported in previous studies conducted in different European countries. Northern rural children scored better in the KTK test battery than their urban peers, similarly to Spanish schoolchildren living in rural areas, who obtained significantly better results in motor competence than children who lived in urban areas (8). Contrarily, Southern urban children showed higher KTK scores than rural children as also reported by Novak et al., who showed that middle school Croatian students living in urban areas had better motor abilities than their rural counterparts (13). It seems that there is not a univocal link between the living setting and the level of motor coordination. Therefore, children's gross motor coordination level and its relationship with living setting is a topic that needs to be better investigated, particularly in Italian context.

The more active children were the Northern children. National data showed that the most of active children attend schools where at least 2 h of weekly PA is performed and where there are initiatives promoting PA (34). Moreover, school playtime could contribute to children's daily PA levels (35). In this perspective, school might play a fundamental role to affect PA level and sedentary behaviors in children. The school environment seems to be the ideal setting for the practice of PA, since it provides opportunities to a great number of children to be physically active during physical education classes and recess (32). Nevertheless, only 34.5% of the classes from the primary schools of the Center of Italy attend at least 2 h of weekly PA, while more than 50% of the classes from the primary schools of the North and the South of Italy attend at least 2 h of weekly PA (www.epicentro.iss.it/okkioallasalute) (5). It seems that Italian schools have some barriers, such as the lack of appropriate areas, equipment, and organized activities during the school day (36), which limit children's opportunities to accumulate PA during the school day. Our urban children of the Center of Italy showed higher PA levels than their rural peers, while children of the South of Italy showed higher PA levels than their urban peers (Figure 3). These conflicting outcomes agreed with other controversial results of PA pattern in rural and urban children and adolescents in the United States (14).

The second aim of the present study was to examine differences in the neighborhood walkability of different school areas from different geographical areas and living settings. The characteristics of neighborhoods were investigated by using Walk Score®, which is a descriptor of the walkability of different areas. Our results showed that the higher proportion of schools in car-dependent neighborhoods were in the Center of Italy. These results were consistent with other Italian reports that showed the low level of walkability in urban areas of the Center of Italy (33). These results would emphasize the criticalities of the neighborhood that limit walkability and could be a basis to support public decisions to intervene in the development of the neighborhoods aimed at encouraging PA. We defined urban or rural setting by population density. However, most rural schools of the present study were in car-dependent neighborhoods where most errands require a car, limiting the use of active transportation such as walking or biking. Therefore, considering the peculiarity of geographical and built environment characteristics of Italy, a new criterion to distinguish urban from rural areas could be introduced based on Walk Score®.

The present study showed the high incidence of overweight and obesity among Italian children. Previous studies revealed that these conditions could lead to health problems such as hypertension, cardiovascular, and metabolic diseases (26, 37). Therefore, to avoid immediate or future health complications, it is fundamental to understand which factors could be related to overweight and obesity in youth. Thus, the last aim of this study was to examine whether motor coordination, PA level, geographical areas, living setting, neighborhood walkability, and gender could predict children's weight status. The multinomial logistic regression results showed that lower MQ, lower PA level, and living in a rural setting were associated with a higher risk for being overweight and/or obese. A Danish study reported similar results showing a significant relationship between body fatness and motor competence (38). A previous Italian investigation reported that lower PA level was associated with a higher risk for being obese (26). The association between rural setting and children's obesity could be due to their lower socio-economic status (28) and therefore to the lower possibility to conduct a correct diet composed by healthy food (29) and to perform organized physical activities (39). It was demonstrated that rural residency was associated with low levels of PA (40). Children's PA levels that could influence children's weight status were often associated with structural influences, such as the physical environment (e.g., access to facilities, safety of neighborhoods, weather conditions) (40). Some environmental investigations showed that neighborhood walkability and the spatial structure of street networks affect PA and weight status condition in children (15, 16). In our study, living in a car-dependent neighborhood was associated with a higher risk for obesity. However, living in walkable areas is not strictly associated with positive walking behaviors (41). This relationship between walkability and BMI categories suggests conducting future studies to investigate the perceived availability of PA opportunities in youth. It might be possible that children perceived barriers to PA even in areas defined as walkable by an objective descriptor such as Walk Score®. According to the theory of functioning and capabilities, well-being is given not only by the simple availability of services and resources of an area, but also by the capability of the population to use them (42). It might be possible that a neighborhood or a region offers infrastructures or recreational areas where children can be active, but they are not able to use them as real resources (42).

Finally, although girls of our study had lower gross motor coordination and PA levels than boys, the logistic regression showed that being a girl was associated with a lower risk for being obese. These conflicting results could be explained by the fact that weight status categories were based on children's BMI. We could speculate that boys had a different body composition from girls, physiologically caused by different hormonal and metabolic factors (43). These factors could characterize boys' body composition by higher lean body mass than girls, explaining their better gross motor coordination performances. It would be necessary to conduct body composition evaluations in future studies to verify it. However, our results were consistent with scientific literature that observed a higher prevalence of overweight and obesity among boys than girls although boys were more active than girls (44), who contrarily showed higher sedentary behaviors than boys (44). Moreover, studies reported gender differences concerning behavioral determinants of overweight and obesity as different eating habits between boys and girls. Girls were more likely to eat healthy than boys, paying more attention to foods, calorie intake and nutrients, and preferring vegetables and fruits with respect to boys (44).

## Strengths and Limitations

The main strength of the study is the large Italian sample recruited. Furthermore, among the strengths, it should be noted that the present work, belonging to a multicenter study (3), is an innovative contribution in understanding the links between children's health-related parameters and urban and rural settings in different Italian regions.

Some limitations to this research should be noted. Since it was a cross-sectional study, causal relationships cannot be inferred. The Central children were from the Lazio region, and the Southern children were from the Sicily region. Future studies should include children from more different regions to generalize the findings of the study to all Italian regions. In addition, we compared the Metropolitan City of Rome with medium-sized cities. Future studies should include cities with similar size and population density. We investigated children's BMI, but we did not have indications regarding their body composition, eating habits, perceived availability of PA opportunities, parental factors, and socio-economic conditions that could influence the weight status. Our data were based on an age group (8–13 years old) that could make difficult to extend our conclusion to younger or older children and adolescents. Finally, walkability was referred as walkability of different school areas. In Italy, primary and secondary schools are very delocalized in the territory, and therefore, we assumed that school address and home address matched (same zip code). Further investigations are needed to verify if children's home address may be a more sensible approach to represent neighborhood walkability.

## Perspective

Globally, Northern children showed better health-related parameters (lower BMI, higher MQ scores, and PA levels) than Central and Southern children, suggesting that Northern children are able to benefit from the available services or interventions. Considering the alarming high percentage of children with motor coordination impairments, targeted PA interventions are mandatory. Moreover, the high percentage of overweight and obese children suggests additional efforts to facilitate an active lifestyle and integrated healthy eating programs in Italian children.

## Data Availability Statement

The raw data supporting the conclusions of this article will be made available by the authors, without undue reservation.

## Ethics Statement

The studies involving human participants were reviewed and approved by the University Ethical Committees of the University of Rome (Rif 5500 Prot. 1070/19), the University of Verona (No. 2019-UNVRCLE-0298910), and the University of Palermo (No. 8/2019), in accordance with the ethical standards laid down in the 1964 Declaration of Helsinki and its later amendments. Additional authorization was provided by school principals/administrators. Written informed consent to participate in this study was provided by the participants' legal guardian/next of kin.

## Author Contributions

MG, SM, CB, and LG: conceptualization and methodology. VB, MG, MG, and LF: data collection. GZ, GB, and MB: data analysis. MG, GZ, ML, FS, and AP: data interpretation. MG and LF: writing—original draft preparation. LG: writing—review and editing. CB and ML: supervision. All authors contributed to the article and approved the submitted version.

## Conflict of Interest

The authors declare that the research was conducted in the absence of any commercial or financial relationships that could be construed as a potential conflict of interest.

## Publisher's Note

All claims expressed in this article are solely those of the authors and do not necessarily represent those of their affiliated organizations, or those of the publisher, the editors and the reviewers. Any product that may be evaluated in this article, or claim that may be made by its manufacturer, is not guaranteed or endorsed by the publisher.
